# Differential Stability of miR-9-5p and miR-9-3p in the Brain Is Determined by Their Unique *Cis*- and *Trans*-Acting Elements

**DOI:** 10.1523/ENEURO.0094-20.2020

**Published:** 2020-06-02

**Authors:** C.K. Kim, A. Asimes, M. Zhang, B.T. Son, J.A. Kirk, T.R. Pak

**Affiliations:** Department of Cell and Molecular Physiology, Loyola University Chicago, Maywood, IL 60153

**Keywords:** degradation, hippocampus, hypothalamus, microRNA, rat, stability

## Abstract

microRNAs (miRs) are fundamental regulators of protein coding genes. In the CNS, miR-9 is highly enriched and critical for neuronal development and function. Mature miRs are derived from a duplex precursor, and the -5p strand (“guide”) is preferentially incorporated into an RNA-induced silencing complex (RISC) to exert its regulatory functions, while the complementary -3p strand (“passenger”) is thought to be rapidly degraded. By contrast, both strands of the miR-9 duplex have unique functions critical for neuronal physiology, yet their respective degradation rates and mechanisms governing degradation are not well understood. Therefore, we determined the degradation kinetics of miR-9-5p and miR-9-3p and investigated the *cis* and *trans* elements that affected their stability in the brain. Using a combination of homogeneous neuronal/astrocyte cell models and heterogeneous brain tissue lysate, we demonstrate the novel finding that miR-9-3p was more stable than the miR-9-5p guide strand in all models tested. Moreover, the degradation kinetics of both miR-9-5p and miR-9-3p were brain-region specific, suggesting that each brain region was differentially enriched for specific degradation factors. We also determined that the 3′ nucleotides harbor important *cis* elements required to not only maintain stability, but also to recruit potential protein degradation factors. We used mass spectrometry to assess the miR-9 interacting proteins and found that the -5p and -3p strands were associated with functionally distinct proteins. Overall, these studies revealed unique miR-9-5p and miR-9-3p degradation kinetics in the brain and proposed critical nucleotide sequences and protein partners that could contribute to this differential stability.

## Significance Statement

miR-9-5p and miR-9-3p are two single-stranded microRNAs (miR) derived from the same RNA duplex that are critical for normal neuronal function. Here, we report the differential degradation kinetics of these neuronally enriched miRs, as well as identify unique regulatory *cis* and *trans* elements that could contribute to the distinct miR-9-5p and miR-9-3p stability in neurons. These findings contribute to the current understanding of how neuronal miRs are degraded and could have functional implications for their respective mRNA targets.

## Introduction

Posttranscriptional regulation of protein-coding genes (mRNA) is a critical mechanism for maintaining cellular homeostasis. Cells must orchestrate a delicate balance between the synthesis of new molecules and the degradation and/or export of older ones. microRNAs (miRs) are a major contributor to this process, as it is estimated that they regulate over 60% of all protein-coding genes in eukaryotic cells (Friedman et al., 2009). The major steps for the biogenesis of miRs have largely been determined; however, the mechanisms of miR degradation are still the focus of ongoing research. Earlier reports suggested that miRs are globally more stable compared with mRNA (Gantier et al., 2011; Rüegger and Großhans, 2012; Zhang et al., 2012), and this stability is thought to be imparted by miR association with RNA binding proteins, such as Argonaute 2 (AGO2). When bound to AGO2, structural analyses dictate that the 5′ and 3′ ends of the mature miR are embedded within the protein, thereby shielding it from potential exoribonucleases (Wang et al., 2008). Recently, mechanisms of target-directed miR degradation (TDMD) have been discovered whereby a highly complementary, endogenous RNA target is capable of dislodging the 3′ end of the miR from the AGO2 PAZ domain, allowing it to be more accessible to *trans* factors responsible for RNA tailing, trimming, and ultimately degradation (Park et al., 2017; Bitetti et al., 2018; Ghini et al., 2018; Kato, 2018; Wightman et al., 2018). The reported mechanisms of TDMD suggest that *cis* sequence motifs of the miRs, as well as the recruitment of *trans* acting proteins to the site of degradation, are crucial determinants of miR degradation kinetics; however, the specifics of these factors remain elusive.

To further add to the complexity of miR degradation, miRs exhibit varying half-lives between different tissues and cell types within an organism (Li et al., 2013). For example, miR stability in the CNS is a striking exception to the long half-lives generally observed in peripheral organs. Neuronal miRs are highly unstable and can be regulated by neuronal activity, suggesting that their silencing function is temporally controlled by external stimuli (Krol et al., 2010; Fu et al., 2016). Indeed, a variety of chemical and electrical stimuli has been shown to dramatically alter miR expression levels in cultured neurons (for review, see Sim et al., 2014), adding another layer of regulation to the unstable nature of neuronal miRs. Notably, the half-life of one of the most abundant neuron-enriched miRs, miR-9-5p, was reported to be <1 h in primary neocortical cells (Sethi and Lukiw, 2009). However, the degradation kinetics of its duplex counterpart, miR-9-3p, was not considered in this study. miR-9-5p is designated as the “guide” strand in most deuterostomes, and its annotation is derived from the mature miR sequence being embedded in the 5′ stem of the miR-9 precursor; conversely, miR-9-3p, or the “passenger” strand, is embedded in the 3′ stem. For most miRs, it is generally accepted that the guide strand of the duplex is preferentially loaded onto AGO2 and is the functionally relevant strand, while the passenger strand is quickly degraded. However, both miR-9-5p and miR-9-3p are neuron-enriched, and their individual functional contributions have been extensively described in regulating critical neuronal processes such as driving neuronal differentiation, initiating angiogenesis, and modulating synaptic plasticity (Yuva-Aydemir et al., 2011; Coolen et al., 2013; Sun et al., 2013; Giusti et al., 2014; Richner et al., 2015; Sim et al., 2016; Madelaine et al., 2017). The significance of their individual functions implies that both the -5p and -3p transcripts must be relatively stable and separately loaded onto AGO2; however, the degradation kinetics of these two miRs, especially miR-9-3p, are not well characterized in the literature. Therefore, the studies herein focused on discerning the relative degradation kinetics of miR-9-5p and miR-9-3p due to their neuronal enrichment and their significant roles in regulating neuronal physiology.

The objective of this study was to determine the relative degradation kinetics and the factors that govern miR-9-5p and miR-9-3p degradation specifically in the rat hypothalamus and ventral hippocampus (vHIPP): regions of the brain that regulate homeostasis in a variety of physiological processes. Moreover, previous studies showed that miR-9-5p and miR-9-3p expression increased with advanced age in these brain regions (Rao et al., 2013). Therefore, in this study, we investigated miR-9 degradation kinetics in aged rats (18 months) when expression levels were highest, underscoring the possibility that the kinetics of miR turnover would be functionally relevant. We hypothesized that the degradation kinetics of miR-9-5p in the aged hypothalamus would be distinct from that of miR-9-3p. Moreover, we predicted that any differences in miR degradation kinetics would be due to intrinsic differences in nucleotide composition (i.e., *cis* factors) or through differential recruitment of *trans* factors that contribute to degradation processes. These possibilities were tested *in vivo* using a rat model and *in vitro* using hypothalamic-derived neuronal cells lines. Collectively, our results indicate that miR-9-5p stability is distinct from that of miR-9-3p, and this difference can be explained, in part, by their unique *cis* and *trans* elements.

## Materials and Methods

### Animals

Female rats (Wistar, five months old; Fisher344, 18 months old, NIH aging colony) were obtained from Charles River Laboratories. Previous studies showed that miR-9-5p and miR-9-3p steady-state expression increased with age in the rat hypothalamus and vHIPP (Rao et al., 2013). Therefore, in this study, we used old (18 month) Fisher344 rats. Rats were pair-housed on arrival and allowed to acclimate for one week before further experimentation. Rats were supplied with standard rat chow and tap water *ad libitum*, and animals were kept on a 12/12 h light/dark cycle with zeitgeber time (ZT) 0 at 7 A.M. All animal protocols were approved by Loyola University Chicago Animal Care and Use Committee (IACUC, permit 2009018). Experiments were conducted in accordance to the guidelines set forth by the IACUC, and all appropriate measures were taken to minimize pain and suffering.

### Cell culture

IVB cells, a neuronal cell line derived from rat hypothalamus (provided by John Kaskow, University of Cincinnati), were grown to 70–80% confluency in DMEM media containing glucose, L-glutamine, sodium pyruvate, and 10% fetal bovine serum (FBS). Cells were subsequently lysed using a 0.5% NP40 buffer with protease and phosphatase inhibitors (Thermo Fisher Scientific, #PI88669). Following cell lysis, protein concentration was determined using a bicinchoninic acid (BCA) assay according to manufacturer instructions (Thermo Fisher Scientific, #23225).

### Actinomycin treatment

IVB cells were treated with actinomycin D (Sigma-Aldrich, A9415) at a final concentration of 10 μg/ml for 2 h to inhibit transcription. Actinomycin D was diluted in dimethyl sulfoxide at a stock concentration of 1 mg/ml before addition to cell culture media. Cells were lysed at three different time points: T0, 15 min, and 60 min. These experiments were performed independently using five different cell passages.

### RNA isolation and cDNA synthesis

Total RNA was isolated from IVB cells using the Zymogen DirectZol kit; 1.0 μg of RNA was reversed transcribed using the Norgen miRNA cDNA Synthesis kit (#54410) according to manufacturer instructions.

### Reverse transcription quantitative polymerase chain reaction (RT-qPCR)

RT- qPCR for miR-9-5p and -3p was performed using forward primers specific to the mature sequence and a Universal Reverse Primer provided by the Norgen miRNA cDNA Synthesis kit (#54410). All reactions were performed in triplicate; 18s rRNA was used as a loading control to normalize the data for ΔΔCt analysis. The following cycling conditions were used: (1) 95°C for 10 min, (2) 95°C for 15 s, (3) 59°C for 20 s, and (4) 72°C for 12 s, and melting curve analysis.

### Primary astrocytes

Primary astrocytes were obtained from the cortex of five-month-old, female Wistar rats (*N* = 3). Cortical tissue was digested using 0.25% trypsin-EDTA, and neuronal cell growth was inhibited with the addition of DMEM: F12 media supplemented with 0.1 mg/ml Primocin following the plating procedure. Primary astrocytes were grown to 70–80% confluency in the following astrocyte medium: DMEM 50:50 F12 media containing glucose, L-glutamine, sodium pyruvate, and 10% FBS.

### *In vivo* tissue preparations

Eighteen-month-old female Fisher 344 rats were killed (*N* = 3), and whole brain was isolated and flash frozen in 2-methylbutane at −30°C. Flash frozen brains were sectioned at 200 μm on a freezing microtome, and regions of interest were microdissected using a 0.75-mm Palkovit’s brain punch tool [Stoelting Co; preoptic area (POA; –0.26 to −1.4 mm relative to bregma), supraoptic nucleus (SON; –0.8 to −3.14 mm relative to bregma), vHIPP (−4.16 to −5.8 mm relative to bregma)] using *The Rat Brain in Stereotaxic Coordinates* (Academic Press, 1986) as a reference. Brain tissue lysate was prepared as described above. Selected samples from the SON brain tissue lysate were treated with 250 μg/ml proteinase K (ThermoFisher Scientific, #25-530-049) and incubated for 1 h at 60°C before use in the miR degradation assay.

### miR degradation assay

The miR degradation assay was adapted for tissue and whole cell lysates based on methods described by Chatterjee and Grosshans (2009). Briefly, oligonucleotide constructs were synthesized with the exact nucleotide sequence of the mature transcript for miR-9-5p and miR-9-3p, [UCUUUGGUUAUCUAGCUGUAUGA] and [AUAAAGCUAGAUAACCGAAAGU], respectively (Integrated DNA Technologies). These single stranded oligonucleotide sequences were then radiolabeled on the 5′ end using [γ32P] ATP (3000 Ci/mmol; PerkinElmer); 10 fmol of the newly radiolabeled sequence was then incubated with 20 μg of protein from either the IVB cell lysate or brain tissue lysate prepared as described above. Incubation of the radiolabeled miR with the lysate was terminated at five different time points by boiling at 95°C for 2 min following the addition of 2× RNA Loading Dye (New England Biolabs, #B0363S). The resulting mixture was then resolved on an 8% urea gel by electrophoresis. Finally, the gel was visualized by phosphoimaging (GE Healthcare, Typhoon) to detect levels of the radiolabeled miR at the various time points. Gel bands were quantified using densitometry analyses with ImageJ (RRID:SCR_003070) software and averaged densitometry values from multiple replicates were plotted on a scatterplot using OriginLab software. Degradation kinetics were determined using a best-fit exponential decay function.

### AGO2 immunoprecipitation

A total of 20 μg of IVB protein lysate was premixed with anti-AGO2, anti-AGO4, β-actin, or IgG antibody (Wako #22023, RRID:AB_1106837; Cell Signaling #6913, RRID:AB_10828811; Cell Signaling #4970, RRID:AB_2223172; Millipore #12-370, RRID:AB_145841) overnight at 4°C. The preformed antibody-antigen complex was then added to 50 μl of PureProteome Protein A/G Magentic Beads (Millipore, #LSKMAGAG02) and incubated for 30 min at room temperature. Next, 10 fmol of radiolabeled miR was added to this mixture and incubated at 37°C for 15 min. The Pureproteome magnetic stand was used to capture the beads, and three washes were performed with PBS containing 0.1% Tween 20 detergent. After the last wash, the bound protein complexes were eluted with the addition of 0.5% NP40 buffer and 2× RNA Loading Dye (New England Biolabs, #B0363S) followed by boiling at 95°C for 2 min.

### RNA immunoprecipitation

A total of 500 μg of vHIPP lysate, 1 μg of biotinylated RNA (Integrated DNA Technologies), 10× protease inhibitor (ThermoFisher Scientific, #PI88669), 40 U/μl RNase inhibitor (ThermoFisher Scientific, #10-777-019), and 2× TENT buffer (20 mm Tris-HCl, 2 mm EDTA, 500 mm NaCl, and 1% Triton X-100) were mixed and incubated at room temperature for 30 min. RNA-protein interactions were fixed using formaldehyde at a 1% final concentration for 10 min; 50 μl of Dynabeads MyOne Streptavidin C1 (ThermoFisher Scientific, #65002) was then added to the above mixture and incubated for another 30 min at room temperature. This mixture was placed on a PureProteome magnetic stand (Millipore Sigma, #LSKMAGS08), and the beads were washed three times with 1× TENT buffer before final elution with 40 μl of 1× Laemmli buffer (Bio-Rad, #161-0747) at 95°C for 5 min. The eluted proteins were then run on a 10% SDS-PAGE gel at 120 mV for 60 min, and proteins were visualized by Coomassie staining (R250).

### In gel digestion

Protein bands were cut into 1-mm^3^ pieces and washed with 100 mm ammonium bicarbonate shaking at 600 rpm for 15 min at 37°C. The gel pieces were then washed with a 50:50 100 mm ammonium bicarbonate and acetonitrile solution at 600 rpm for 15 min at 37°C. The final wash was performed with 100% acetonitrile at the same conditions as the previous washes. The gel pieces were reduced with 250 mm DTT (Sigma-Aldrich, #D9779-5G) at 550 rpm for 60 min at 56°C and subsequently alkylated with 50 mm iodoacetamide (Sigma-Aldrich, #A3221-10VL) in the dark for 45 min at room temperature. Finally, 2 μg of MS grade Trypsin (ThermoFisher Scientific, #PI90057) was added to the gel pieces to digest the protein overnight at 37°C while shaking at 600 rpm.

### Mass spectrometry

The analysis was conducted on a Dionex Ultimate 3000 RSLCnano coupled to a LTQ Orbitrap XL (ThermoFisher Scientific). A total of 10 μl of each sample was injected onto the column. Mobile phase A was 100% H_2_O, 0.1% formic acid, and mobile phase B was 80% ACN, 0.08% formic acid. The flow rate was set at 300 nl/min. The column oven was set at 40°C. A gradient of 5–45% mobile phase B was run over 105 min followed by a wash cycle and equilibration of the column. Total run time on the HPLC was 138 min. An EASY-Spray column (2-μm particle size, 25 cm × 75 μm ID, PepMap C18) was used to separate the peptides, and an EASY-Spray ionization source was used for ionization. Data-dependent acquisition was conducted with the mass spectrometer. The first scan was recorded with the Orbitrap followed by 10 subsequent ion trap scans (FT-IT detection) on the top 10 most abundant ions. Collision-induced dissociation (CID) was used as the activation source with normalized collision energy at 35. Charge state rejection was enabled for +1 charged ions, and dynamic exclusion was enabled for a list size of 500 over 30 s. The data were analyzed using PEAKS 8.5 software.

### Statistics

All statistical analysis was performed using OriginLab software. A two-sample *t* test was performed to compare the mean half-lives of miR-9-5p and miR-9-3p in cell lines. The actinomycin D experiments were analyzed using a two-way ANOVA with time and miR construct as the two factors. The brain region-specific degradation of miR-9-5p and -3p was also analyzed using a two-way ANOVA with brain region and miR construct as the two factors. A one-way ANOVA was performed to compare the mean densitometry values of the various miR-9-5p constructs at the T1 time point. A Tukey’s *post hoc* test was subsequently performed to determine statistically significant differences between group means. All data points are displayed as mean ± SEM, and statistical significance was noted when *p* < 0.05.

## Results

### The passenger strand miR-9-3p was more stable than the guide strand miR-9-5p in a hypothalamic cell line

**Figure 1.**
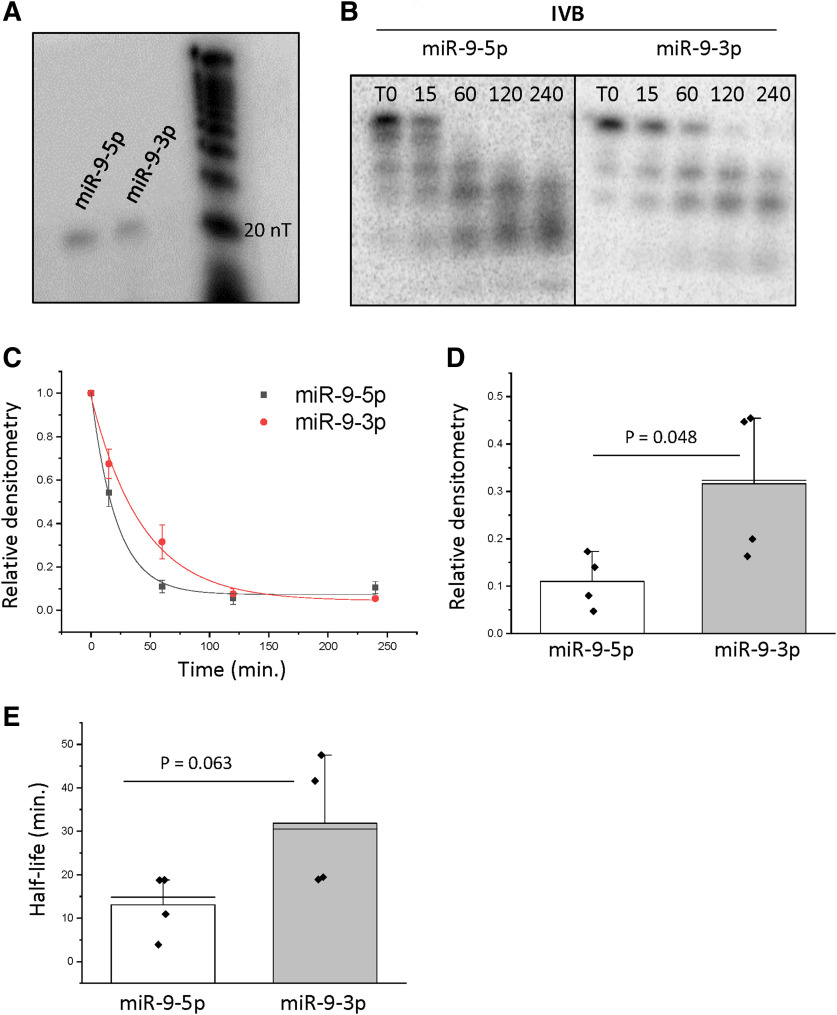
miR-9-3p was more stable than miR-9-5p in a hypothalamic cell line. ***A***, Gel electrophoresis of ^32^P-labeled miR-9-5p and miR-9-3p showing bands at their correct size: 23 and 22 nucleotides (nT), respectively. ***B***, Representative gel image of miR-9-5p and miR-9-3p degradation over time (minutes) following incubation in hypothalamic-derived neuronal cell (IVB) lysate for 0, 15, 60, 120, or 240 min. ***C***, Scatterplot of normalized densitometry values analyzed from gel images and fit with an exponential decay function (black line = miR-9-5p; red line = miR-9-3p; *N* = 4/group). ***D***, Normalized densitometry values at the 60-min time point for miR-9-5p and miR-9-3p. Data are represented as mean ± SEM (*N* = 4/group). ***E***, Mean half-lives of miR-9-5p and miR-9-3p derived from best-fit exponential decay functions. Results are represented as mean ± SEM (*N* = 4/group), and horizontal line overlaying the bar graph indicates the median. Data were analyzed using a two-sample *t* test.

We used a basic biochemical approach to determine whether the unique nucleotide sequence of miR-9-5p and miR-9-3p affected their degradation kinetics when exposed to identical cellular lysate components. Importantly, to our knowledge, this assay has only been done previously in cell-free conditions making this the first study to observe the degradation products of these miRs following exposure to native cell and whole tissue constituents. First, ^32^P-labeled oligonucleotides identical to each miR were incubated in cell lysate purified from hypothalamic-derived neuronal cells (IVB). The cells were subjected to a mild lysis buffer that was not likely to disrupt the nuclear envelope, as we hypothesized that most miR degradation factors would reside in the cytoplasm. Each construct was first visualized at the correct size compared with a nucleic acid ladder size marker (Fig. 1*A*). Next, radiolabeled miR-9-5p and miR-9-3p were incubated in IVB lysate for five different time points (T0, 15, 60, 120, 240 min). Our results showed that miR-9-3p, canonically considered the passenger strand, was more stable than miR-9-5p (Fig. 1*B*,*C*). Approximately 50% of the full-length miR-9-5p signal was reduced at the 15-min time point, and by the 1-h time point, the full-length signal was completely abolished. Comparatively, the signal corresponding to full-length miR-9-3p was significantly higher than miR-9-5p at the 1-h time point, indicating slower degradation kinetics (*p* = 0.048; Fig. 1*B*,*D*). A comparison of the mean half-lives derived from the best-fit exponential decay functions indicated that miR-9-3p was approximately twice as stable as miR-9-5p, exhibiting half-lives of 31.85 and 13.09 min, respectively (Fig. 1*C*,*E*). Importantly, a small fraction of both radiolabeled constructs (miR-9-5p and miR-9-3p) was shown to bind AGO2, but not AGO4 or β-actin, demonstrating that the radiolabeled miRs bind specifically to relevant RNA binding proteins in this assay (Extended Data Fig. 1-1).

10.1523/ENEURO.0094-20.2020.f1-1 Extended Data Figure 1-1 AGO2 binds to ^32P^-labeled miR-9-5p and miR-9-3p. Representative gel image of ^32P^-labeled miR-9-5p, miR-9-3p, and an equimolar equivalent mixture of both following immunoprecipitation with (***A***) AGO2, (***B***) IgG control, and (***C***) AGO4 and β-actin. Download Figure 1-1, TIF file

**Figure 2.**
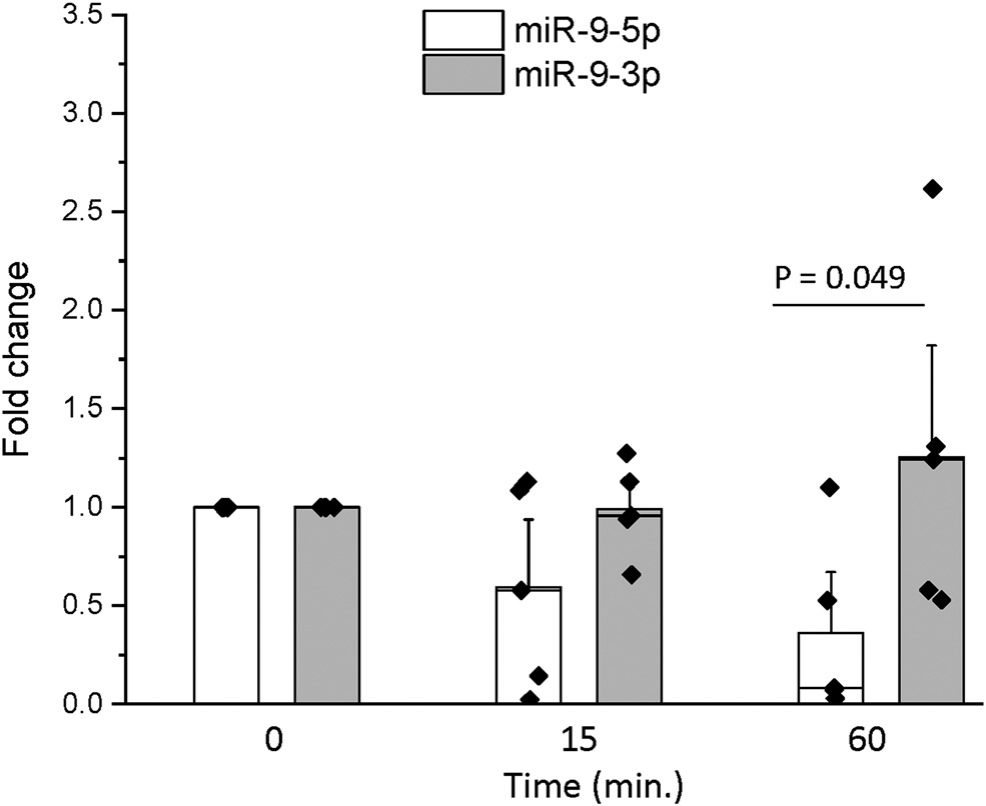
miR-9-3p was more stable than miR-9-5p following transcriptional inhibition. Hypothalamic-derived IVB cells were treated with the transcriptional inhibitor actinomycin D for 2 h. Cells were lysed at 0, 15, and 60 min following treatment, total RNA was isolated, and RT-qPCR was performed for miR-9-5p and miR-9-3p (*N* = 5/group). Results were analyzed using the ΔΔCt method and are represented as mean fold change ± SEM, and horizontal line overlaying the bar graph indicates the median. Data were analyzed by two-way ANOVA with time and miR construct as factors. A Tukey’s *post hoc* test was performed to determine statistically significant differences between group means.

We also observed size differences in the miR-9-5p and miR-9-3p degradation products (i.e., smaller size bands on the gel), suggesting that the miRs were cleaved at different locations. Specifically, miR-9-5p had four discrete bands corresponding to the degradation products, whereas miR-9-3p only had 2 discrete bands (Fig. 1*B*). Next, we used a second method to verify that miR-9-3p was more stable by measuring the degradation rates of endogenously expressed mature miR-9-5p and miR-9-3p following transcriptional inhibition with actinomycin D. Consistent with the miR degradation assays, these experiments also showed that miR-9-3p was more stable than its miR-9-5p counterpart (Fig. 2). Two-way ANOVA analysis revealed that there was a significant main effect of miR construct (Table 1). Subsequent *post hoc* analyses using Tukey’s HSD revealed that at the 1-h time point, miR-9-3p levels were significantly higher (*p* = 0.049) compared with miR-9-5p, indicating a more stable profile (Fig. 2). Notably, this assay used intact live cells and confirmed that the relative stability of miR-9-3p compared with miR-9-5p was similar to the purely biochemical approach of the miR degradation assay described above.

**Table 1:** Statistical Analysis

Dataset	Data structure	Type of test	Power
Fig. 1*D*	Normal distribution	Two-sample *t* test	*p* = 0.048
Fig. 1*E*	Normal distribution	Two-sample *t* test	*p* = 0.063
Fig. 2	Normal distribution	Two-way ANOVA Tukey’s HSD test	Time: *F*_(1,24)_ = 0.646, *p* = 0.533
			miR: *F*_(1,24)_ = 6.724, *p* = 0.016
			Time × miR interaction: *F*_(1,24)_ = 2.414, *p* = 0.111
Fig. 3*C*	Normal distribution	Two-way ANOVA Tukey’s HSD test	Brain region: *F*_(1,12)_ = 5.543, *p* = 0.020
			miR: *F*_(1,12)_ = 100.365, *p* = 3.511 × 10^-7^
			Brain region × miR interaction: *F*_(1,12)_ = 4.979, *p* = 0.027
Fig. 5*C*	Normal distribution	Two-sample *t* test	*p* = 1.658 × 10^–4^
Fig. 6*D*	Normal distribution	One-way ANOVA	*F*_(2,9)_ = 10.119, *p* = 0.005
Fig. 6*E*	Normal distribution	Two-sample *t* test	*p* = 0.045

### miR-9-3p was more stable than miR-9-5p in a brain region-dependent manner

**Figure 3.**
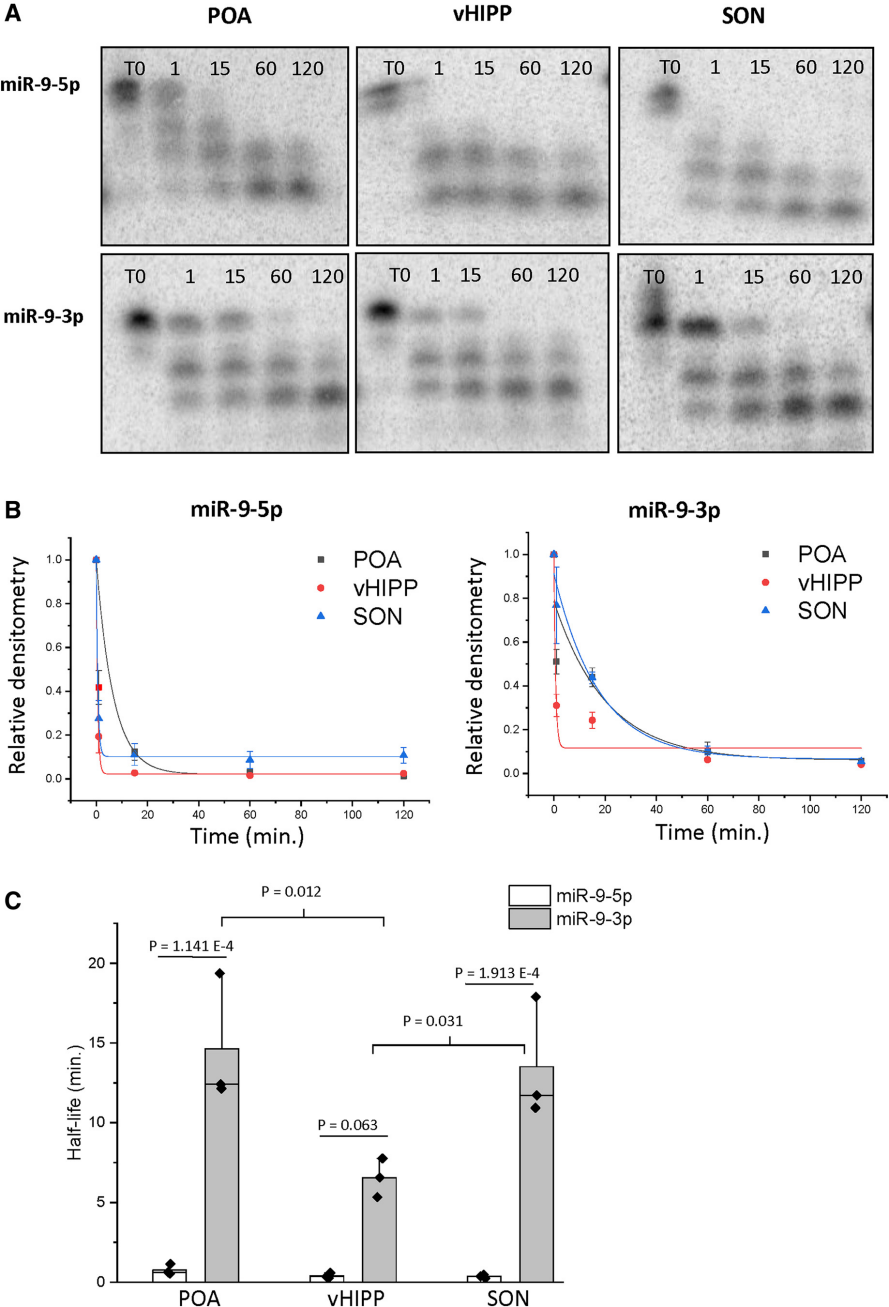
miR-9-3p was more stable than miR-9-5p in a brain region-dependent manner. ***A***, Representative gel image of ^32^P-labeled miR-9-5p and miR-9-3p following incubation with rat brain lysate (*N* = 3/brain region). ***B***, Scatterplot of normalized densitometry values analyzed from gel images and fit with an exponential decay function showing brain region-specific degradation kinetics of miR-9-5p and miR-9-3p. ***C***, Mean half-lives of miR-9-5p and miR-9-3p in various brain regions derived from best-fit exponential decay functions. Results are represented as mean ± SEM (*N* = 3/group), and horizontal line overlaying the bar graph indicates the median. Data were analyzed by two-way ANOVA with brain region and miR construct as factors. A Tukey’s *post hoc* test was performed to determine statistically significant differences between group means.

Next, the degradation kinetics of miR-9-5p and miR-9-3p were investigated using brain tissue lysate from three different rat brain regions: the POA, the vHIPP, and the SON. These regions were selected due to previous reports demonstrating endogenous miR-9-5p and miR-9-3p expression in these regions (Rao et al., 2013, 2015). Unexpectedly, the time course for both miR-9-5p and miR-9-3p degradation in each of these brain regions was consistently faster than what was observed in the hypothalamic IVB neuronal cell lines, although total protein concentrations were identical in the different lysates (Fig. 3*A*,*B*). The signal corresponding to the full-length miR-9-5p disappeared almost immediately on exposure to brain tissue lysate; therefore, T0 had to be redefined using lysis buffer alone to determine the correct starting concentration, and the time scales were adjusted accordingly to T0, 1, 15, 60, and 120 min (Fig. 3*A*). Our results also showed brain region-specific differences in miR-9-5p degradation. For instance, the signal corresponding to full-length miR-9-5p was present at the 1-min time point in POA lysate, but absent in vHIPP and SON lysate (Fig. 3*A*). A two-factor ANOVA analysis of miR half-lives showed that there was a statistically significant main effect of brain region (factor 1) and a significant main effect of miR construct (factor 2). There was also a statistically significant interaction between brain region and miR construct, demonstrating that the degradation rate of each miR was dependent on brain region (Table 1). Consistent with the data observed in the IVB cell line, the half-life of miR-9-3p was significantly longer in all three brain regions compared with miR-9-5p (Fig. 3*C*). Full-length miR-9-3p persisted to the 15-min time point in every brain region. However, differential degradation kinetics for miR-9-3p were still observed dependent on brain region, as miR-9-3p exhibited a longer half-life in the SON and POA compared with the vHIPP (Fig. 3*C*). These data suggest that miR degradation might occur rapidly in the intact rat brain, but there are also distinct region-dependent kinetics.

### Rapid miR-9-5p degradation in vivo was dependent on protein concentration of the lysate

**Figure 4.**
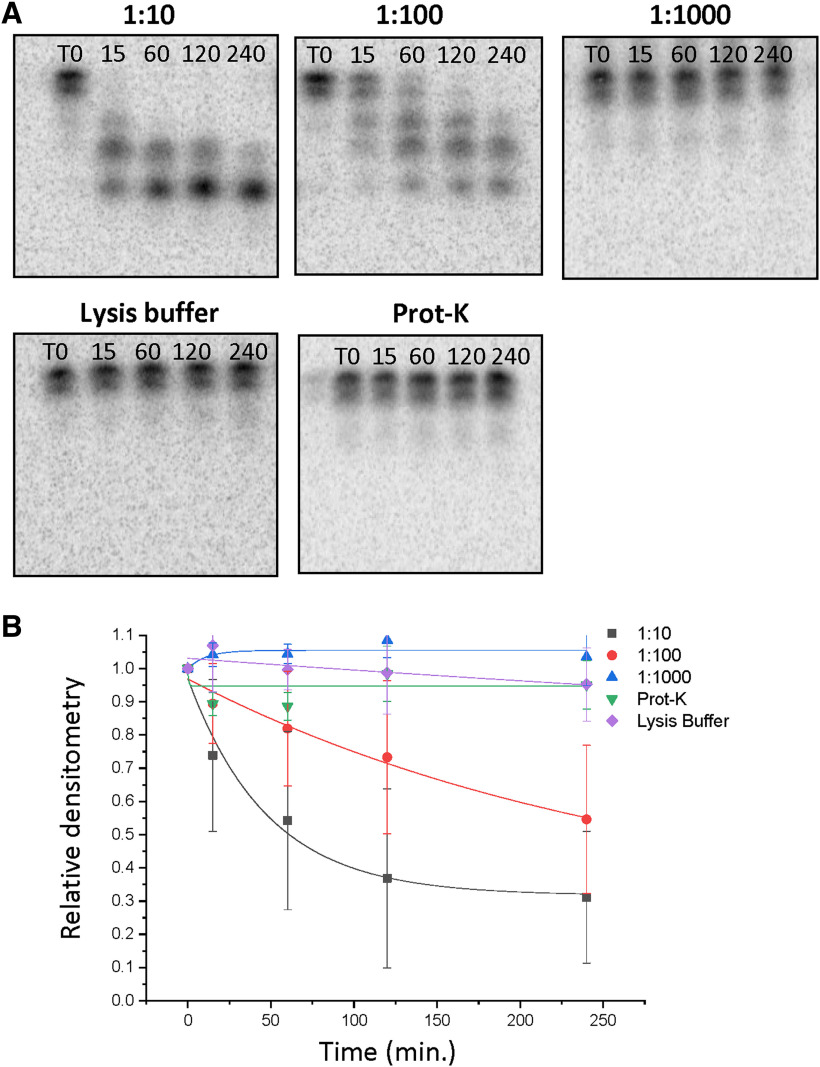
Rapid miR-9-5p degradation in rat brain (SON) tissue lysate was dependent on protein concentration of the lysate. ***A***, Representative gel image of ^32^P-labeled miR-9-5p following incubation with 1:10, 1:100, and 1:1000 dilutions of rat brain (SON) lysate, lysis buffer alone, or following SON lysate treatment with proteinase K for 60 min at 60°C. ***B***, Scatterplot of normalized densitometry values analyzed from gel images and fit with an exponential decay function showing concentration-dependent degradation kinetics of miR-9-5p (*N* = 4/group).

The rapid degradation of miR-9-5p and miR-9-3p that was observed in the brain tissue lysate suggested that there could be enriched levels of specific miR degradation factors compared with the cell lysate. Therefore, to determine whether rapid degradation of miR-9-5p adhered to first order degradation kinetics, radiolabeled miR-9-5p was incubated in various dilutions of SON lysate (1:10, 1:100, 1:1000) for the miR degradation assay. The SON was used for these experiments, because this was the region where miR-9-5p exhibited the fastest degradation (Fig. 3*B*). The results showed a steady increase in miR-9-5p stabilization on increased lysate dilution, culminating in the complete elimination of any degradation products after 4 h of incubation at a dilution of 1:1000 (Fig. 4*A*,*B*). We also hypothesized that the unknown miR degradation factors present in the brain tissue lysate were proteins, and not nucleic acids, steroids, or other chemical compounds. To test this hypothesis, we performed the same assays using lysis buffer alone (i.e., no lysate present) and compared that to proteinase K-treated SON lysate (Fig. 4*A*). Our results demonstrated that miR-9-5p was stable across all time points in both of these treatment groups, suggesting that an unknown protein of fairly high abundance was present in the brain tissue lysate and likely contributed to rapid miR-9-5p degradation.

### Degradation of miR-9-5p was slower in primary astrocytes compared with whole brain tissue lysate

**Figure 5.**
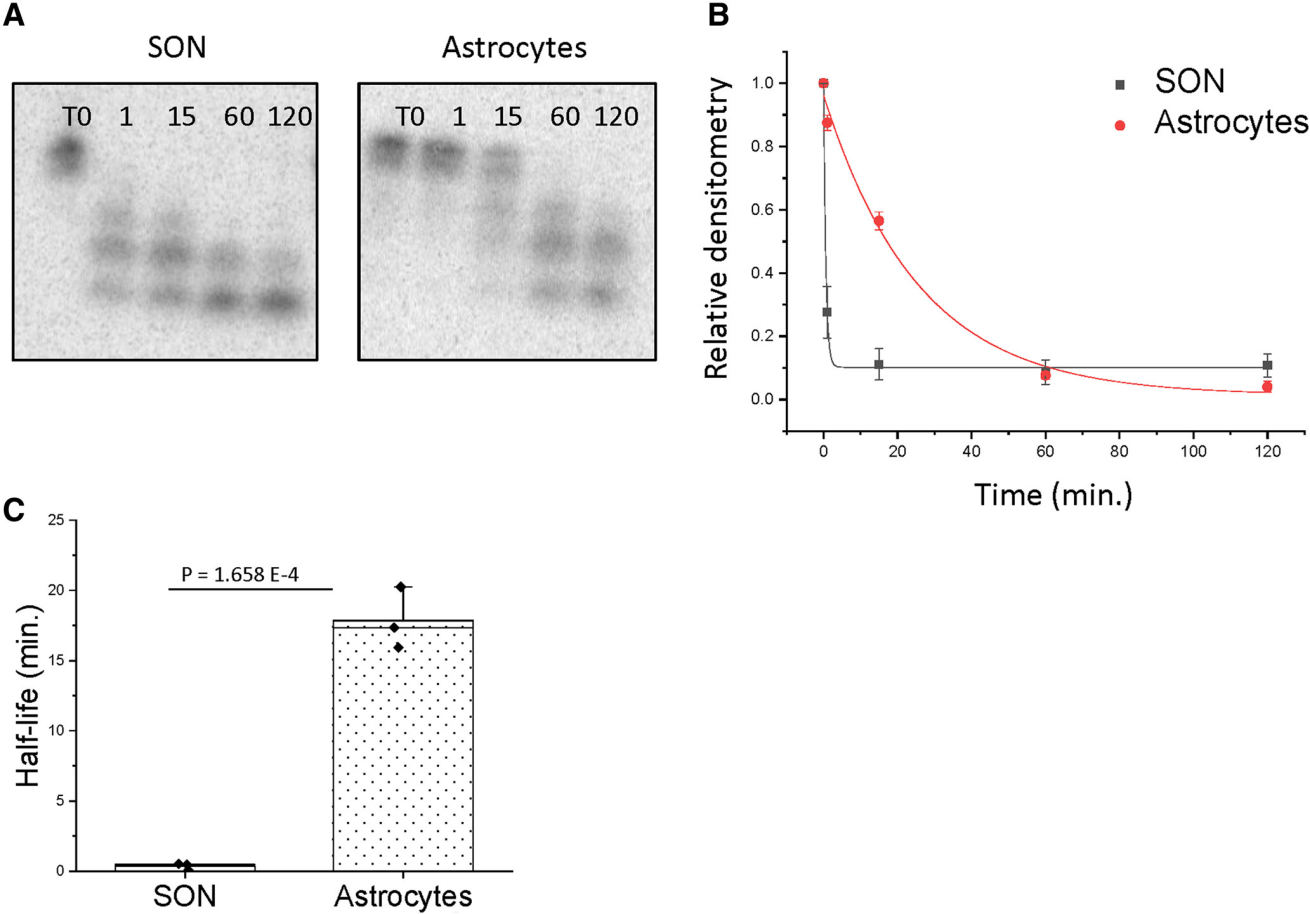
Degradation of miR-9-5p was slower in primary astrocytes compared with rat brain (SON) tissue lysate. ***A***, Representative gel image of ^32^P-labeled miR-9-5p following incubation with rat brain tissue (SON) lysate or rat primary astrocytes for 0, 1, 15, 60, or 120 min (*N* = 3). ***B***, Scatterplot of normalized densitometry values analyzed from gel images and fit with an exponential decay function. ***C***, Mean half-lives of miR-9-5p were derived from best-fit exponential decay functions. Results are represented as mean ± SEM (*N* = 3/group), and horizontal line overlaying the bar graph indicates the median. Data were analyzed using a two-sample *t* test.

Brain tissue lysate is a heterogeneous cell population consisting primarily of neurons and various types of glial cells. We predicted that the glial contribution might account for the enhanced rate of degradation observed in brain tissue lysate compared with the slower rate observed in the homogenous neuronal cell lysate (i.e., IVB cells). Therefore, we used primary astrocytes isolated from the rat brain to determine the contribution of glial cells to miR-9-5p degradation. Contrary to our prediction, miR-9-5p degradation kinetics were relatively stable when incubated with astrocyte only lysate. Specifically, the full-length miR-9-5p signal could be detected at the 15-min time point (Fig. 5*A*,*B*), suggesting that there are inherent differences in miR degradation kinetics between immortalized neuronal cell lines and neurons from the rat brain. Further, the average half-life was 17.84 min closely resembling the profile we observed with the neuronal cell lysate (IVB cells; Fig. 5*C*).

### Nucleotide sequences at both the 5′ and 3′ end contribute to miR-9 stability

**Figure 6.**
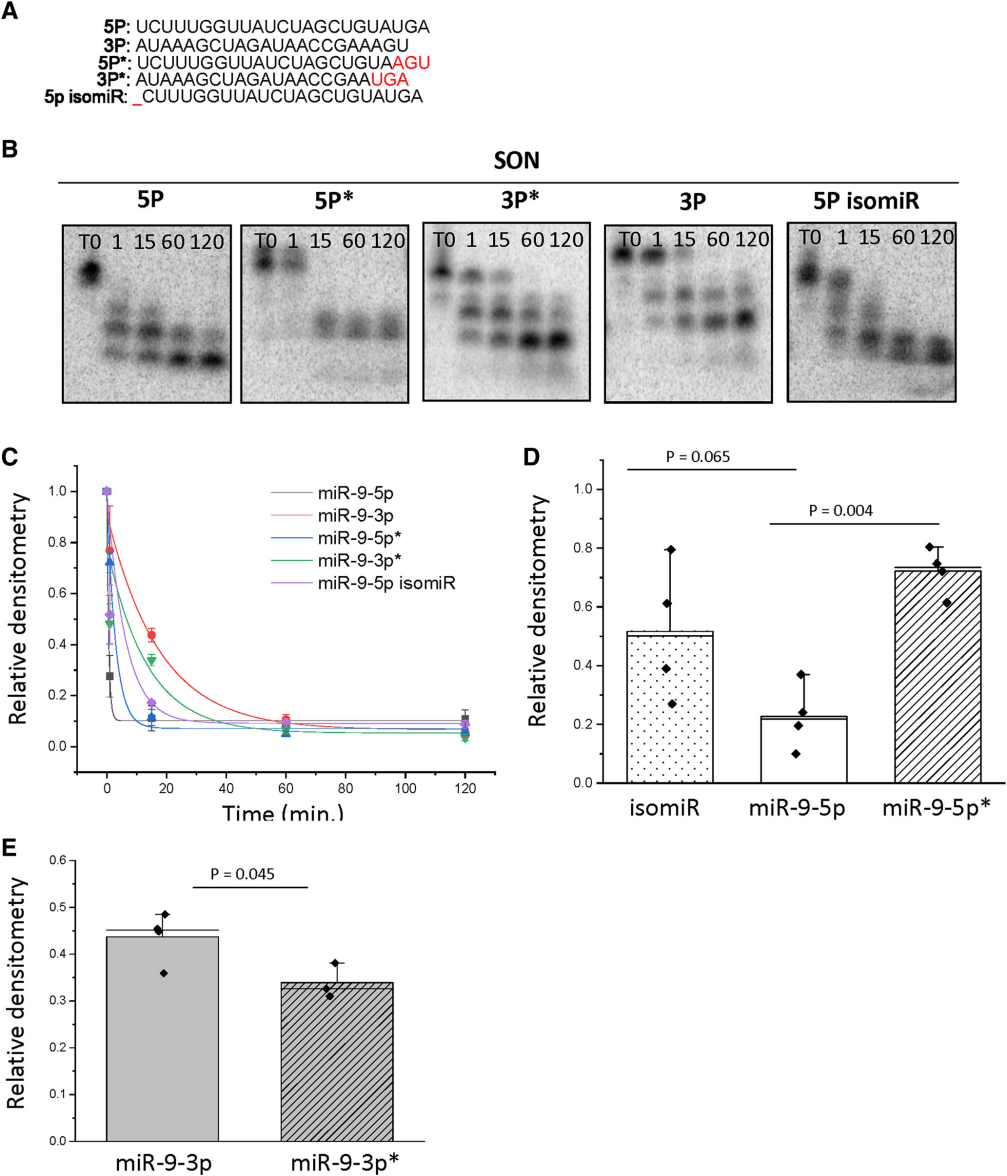
Nucleotide sequences at both the 5′ and 3′ end contribute to miR-9 stability. ***A***, Schematic diagram showing the nucleotide compositions of the miR-9 constructs: 5P = full-length canonical miR-9-5p, 3P = full-length canonical miR-9-3p, 5P* = miR-9-5p with a UGA to AGU substitution at the 3′ end, 3P* = miR-9-3p with an AGU to UGA substitution at the 3′ end, 5p isomiR = miR-9-5p with a U deletion at the 5′ end. Red font indicates nucleotide variation from canonical sequences. ***B***, Representative gel image showing the degradation kinetics of ^32^P-labeled miR-9-5p, -3p, 5p*, 3p*, and 5p isomiR following incubation in rat brain (SON) lysate for 0, 1, 15, 60, or 120 min. ***C***, Scatterplot of normalized densitometry values analyzed from gel images and fit with an exponential decay function showing that both 5′ and 3′ end modifications affect stability (*N* = 3–4/group). ***D***, Normalized densitometry values at the 1-min time point for the miR-9-5p, 5p*, and isomiR constructs. Results are represented as mean ± SEM (*N* = 3–4/group). Data were analyzed by one-way ANOVA with miR construct as the categorical factor. A Tukey’s *post hoc* test was performed to determine statistically significant differences between group means. ***E***, Normalized densitometry values at the 15-min time point for miR-9-3p and -3p*. Results are represented as mean ± SEM (*N* = 3–4/group), and horizontal line overlaying the bar graph indicates the median. Data were analyzed using a two-sample *t* test.

One possible explanation for the observed degradation differences in miR-9-5p and miR-9-3p was the distinct nucleotide sequences (i.e., *cis* factors) of each miR, especially considering that differences in degradation rates and cleavage products occurred despite both miRs being exposed to identical lysate conditions. Moreover, the presence of multiple degradation products for miR-9-5p and miR-9-3p suggested that at least some of the degradation occurred in a 3′ to 5′ direction, due to the radiolabeled phosphate being located at the 5′ position and subsequent visibility of a smaller radiolabeled products on the gel. Therefore, to test the importance of the 3′ end nucleotide sequence in determining stability, the last three nucleotides of miR-9-5p and miR-9-3p were exchanged to create two new chimeras: 5p* and 3p*. The 5p* chimera had a UGA to AGU substitution at the 3′ end, while the 3p* had an AGU to UGA substitution (Fig. 6*A*). Our results demonstrated that swapping just three nucleotides at the 3′ end of miR-9-5p with miR-9-3p (i.e., the *5p construct) was sufficient to significantly increase the stability of miR-9-5p at 1.0 min when incubated with lysate taken from the SON (*p* = 0.004; Fig. 6*B*,*D*). Conversely, this swap had the opposite effect on miR-9-3p (i.e., the 3p* construct) with it showing significantly faster degradation at 15 min. Compared with its native sequence (*p* = 0.045; Fig. 6*B*,*E*). Therefore, swapping the 3′ end nucleotides caused miR-9-5p to degrade at a more similar rate as miR-9-3p and vice versa, suggesting that this sequence motif directly contributed to miR degradation kinetics (Fig. 6*D*,*E*).

It was evident that some amount of degradation also occurred from the 5′ end, based on a small decrease in the total relative intensity of the gel bands from the initial incubation time point to the final time point. Therefore, we altered miR-9-5p to match that of a documented endogenous isomiR to determine the contribution of 5′ nucleotides to the degradation profile. This isomiR is endogenously generated from alternative Drosha cleavage and results in one less uracil at the 5′ terminal end of the miR (Bofill-De Ros et al., 2019). Interestingly, the miR-9-5p isomiR tended to be more stable than the miR-9-5p annotated as the wild type (*p* = 0.065; Fig. 6*B*,*D*). Together, these data provide evidence that *cis* elements at both the 5′ and 3′ ends of the miR are critical for determining its degradation kinetics.

### miR-9-5p and miR-9-3p were associated with distinct proteins in the vHIPP

**Figure 7.**
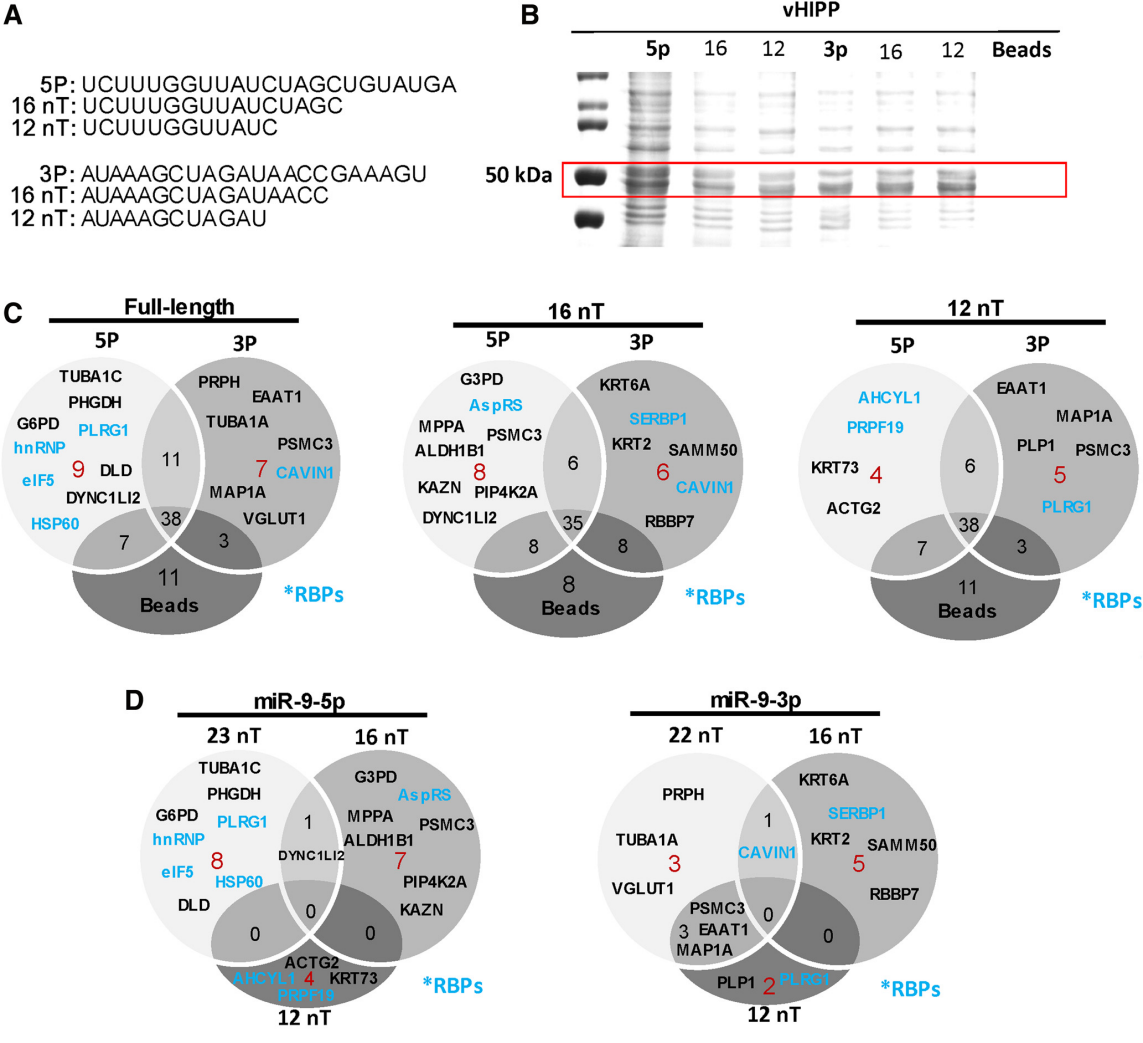
miR-9-5p and miR-9-3p were associated with distinct proteins in the rat vHIPP. ***A***, Various length nucleotides matching the sequence for miR-9-5p (23, 16, 12 nT) and miR-9-3p (22, 16, 12 nT) were modified with a biotin tag on the 5′ end and incubated with lysate from the rat vHIPP. ***B***, Biotinylated miR constructs were purified using streptavidin-coated magnetic beads and resolved on a 10% SDS-PAGE gel followed by Coomassie G-250 staining. Red box indicates gel bands that were dissected for in-gel digestion followed by mass spectrometry. ***C***, Venn diagrams with number of unique proteins identified by mass spectrometry compared between miR-9-5p and miR-9-3p at various sequence lengths plus beads only, or ***D***, comparison of identified proteins within miR-9-5p (23, 16, 12 nT) and miR-9-3p (22, 16, 12 nT). Red color numbers indicate proteins unique to each construct and black numbers indicate proteins common between constructs. Blue lettering indicates known RNA binding proteins.

The contribution of unique *cis* elements to miR degradation rates suggested that these specific nucleotide sequences might recruit different *trans* factors, which then resulted in distinct degradation products for miR-9-5p and miR-9-3p. Our data also demonstrated that miR degradation was dependent on unknown proteins present in the brain tissue lysate (Fig. 4*B*). Therefore, we analyzed the proteins associated with each miR using a discovery-based mass spectrometry approach to determine whether these miRs were associated with distinct proteins that might be involved in RNA degradation processes. The vHIPP was used for the proteomic studies as it represented a relatively large region of the brain compared with smaller specific hypothalamic nuclei, allowing for a sufficient starting concentration of protein. Biotinylated miR-9-5p and miR-9-3p (Fig. 7*A*) were incubated with vHipp lysate, and bound proteins were eluted and run on a 10% SDS-PAGE gel. We focused on identifying proteins from prominent bands present at the 45- to 55-kDa range that was evident upon electrophoresis of proteins following streptavidin-bead purification (Fig. 7*B*). These bands were subsequently digested into smaller peptide fragments and analyzed by mass spectrometry.

We also analyzed the proteins that were associated with the approximated degradation products of miR-9-5p and miR-9-3p in the vHIPP by creating biotinylated sequences that were shorter in length from the 3′ direction (16 and 12 nucleotides each; Fig. 7*A*). These shortened constructs were selected due to the relative stability of the degradation products compared with the full-length transcript. Interestingly, the proteins associated with these shortened constructs were also enriched at the 45- to 55-kDa molecular weight range (Fig. 7*B*). Mass spectrometry analysis revealed full-length miR-9-5p had nine unique associated proteins compared with seven unique proteins for miR-9-3p (Fig. 7*C*; Table 2). The shortened miRs also had distinct proteins associated with them, not only between the -5p and -3p transcripts, but also compared with their own respective full-length transcripts (Fig. 7 *C*,*D*; Table 2). We used Panther gene ontology software to analyze the identified proteins according to their function, and the most represented functional class for full-length miR-9-5p was related to cellular metabolism (∼33%; Table 3). In contrast, the most represented functional class of proteins captured by full-length miR-9-3p was related to cytoskeletal organization and transport (∼57%; Table 3). Moreover, ∼44% of the nine unique proteins identified for miR-9-5p were previously reported to have RNA binding capabilities.

**Table 2:** List of unique proteins that were associated with various length nucleotides matching the sequence for miR-9-5p (23, 16, 12 nT) and miR-9-3p (22, 16, 12 nT), as identified by mass spectrometry using PEAKS software

5P 23 nT
Protein
D-3-phosphoglycerate dehydrogenase
Glucose-6-phosphate 1-dehydrogenase
Heterogeneous nuclear ribonucleoprotein
60-kDa heat shock protein
Eukaryotic translation initiation factor 5
Cytoplasmic dynein 1 light intermediate chain 2
Tubulin α-1C chain
Dihydrolipoyl dehydrogenase
Pleiotropic regulator 1
5P 16 nT
Protein
Glyceraldehyde-3-phosphate dehydrogenase
Aspartate–tRNA ligase cytoplasmic
Mitochondrial-processing peptidase subunit α
Kazrin
Cytoplasmic dynein 1 light intermediate chain 2
26S proteasome regulatory subunit 6A
Phosphatidylinositol 5-phosphate 4-kinase type-2 α
Aldehyde dehydrogenase X
5P 12 nT
Protein
S-adenosylhomocysteine hydrolase-like protein 1
Pre-mRNA-processing factor 19
Keratin Type II cytoskeletal 73
Actin γ-enteric smooth muscle
3P 22 nT
Protein
Peripherin
Excitatory amino acid transporter 1
Microtubule-associated protein 1A
Tubulin α-1A chain
Caveolae-associated protein 1
Vesicular glutamate transporter 1
26S proteasome regulatory subunit 6A
3P 16 nT
Protein
Caveolae-associated protein 1
Keratin Type II cytoskeletal 6A
Plasminogen activator inhibitor 1 RNA-binding protein
Sorting and assembly machinery component 50 homolog
Keratin Type II cytoskeletal 2 epidermal
Histone-binding protein RBBP7
3P 12 nT
Protein
Excitatory amino acid transporter 1
Pleiotropic regulator 1
Microtubule-associated protein 1A
26S proteasome regulatory subunit 6A
Myelin proteolipid protein

**Table 3:** Table summarizing proteins identified by mass spectrometry that were associated with various length nucleotides matching the sequence for miR-9-5p (23, 16, 12 nT) and miR-9-3p (22, 16, 12 nT) and organized by functional class according to Panther gene ontology analysis

	Cytoskeletal	Membrane trafficking	Protein modifying	Transporter	Metabolism	Nucleic acid binding	Translation
miR-9-5p	22.2%	–	–	–	33.3%	11.1%	11.1%
16 nT	12.5%	–	25%	–	37.5%	–	12.5%
12 nT	40%	–	–	–	20%	–	–
miR-9-3p	28.6%	14.3%	14.3%	28.6%	–	–	–
16 nT	–	16.7%	–	–	–	16.7%	–
12 nT	20%	–	20%	20%	–	–	–

Overall, the total number of identified proteins was reduced for each miR as its sequence was progressively shortened. However, the representation of metabolism-related proteins remained high in the proteins associated with both the full-length and the 16-nT (nucleotides) miR-9-5p fragment (Fig. 7*D*; Table 3). Similarly, both the full-length miR-9-3p and its 16-nT fragment was associated with caveolae-associated protein 1 (CAVIN1), a protein implicated in ribosomal RNA synthesis, but this association was lost at the 12-nT length (Fig. 7*D*). The proteins associated with the 12-nT fragment (miR-9-3p) also did not have a dominant functional class, but pleiotropic regulator 1 (PLRG1), a spliceosomal component, was identified as a potential binding partner. The proteins associated with the 12-nT (miR-9-5p) fragment were again most represented in protein classifications pertaining to metabolism and cytoskeletal organization (∼60%), suggesting that miR-9-5p retains proximity to proteins in the same functional milieu despite its degradation (Fig. 7*D*; Table 3). Overall, these data identified potential interactions between the miR-9-5p and miR-9-3p degradome and other RNA processes such as splicing, mRNA processing, and ribosomal RNA synthesis in the rat vHIPP.

## Discussion

The determinants of miR degradation kinetics in the context of the CNS are poorly understood. In the present study, we describe the novel finding that miR-9-3p was more stable than its duplex counterpart, miR-9-5p, when exposed to identical neuronal cell lysates. Our current view of miRs dictates that the mature strand designated as the guide, often derived from the 5′ stem of the pre-miR, is more stable than the strand designated as the passenger, making these data a novel and likely biologically relevant finding. Moreover, our data demonstrated that the relative stability of each strand was dependent on their unique *cis* factor sequence motifs residing at the 3′ end, perhaps through recruitment of distinct degradation factors that are unique to each miR. Indeed, we identified potential *trans* acting factors that were differentially associated with not only full-length miR-9-5p and miR-9-3p, but also to their approximated degradation products. We also showed that the relative abundance of the protein factors contributing to the differential degradation rates of miR-9-5p and miR-9-3p was likely different depending on brain region. Taken together, these data contribute to the current understanding of miR degradation kinetics, specifically in the biologically relevant context of the female rat brain.

Our data are consistent with previous studies suggesting that miRs in the brain are degraded rapidly, underscoring the importance of understanding how these relatively unstable miRs are able to exert functional effects downstream. miR-9-5p and miR-9-3p have repeatedly been shown to be neuron-enriched, and previous studies have demonstrated a negative correlation between miR stability and overall expression (Li et al., 2013). Neuronal miR-9-5p and miR-9-3p seem to follow this pattern: they are both highly expressed and rapidly degraded (Sethi and Lukiw, 2009; Amar et al., 2012), supporting a model whereby there is constitutive production and turnover of these particular transcripts. A potential benefit of such steady state kinetics is readily apparent in the context of neuronal systems in which the cell could quickly respond to dynamic changes in extracellular input, such as changes to synaptic firing frequency. Indeed, an example of activity-induced changes to miR stability was observed in retinal neurons responding to changes to light/dark stimuli (Krol et al., 2010). The relative stability of miR-9-3p that was observed from our data (Figs. 1*D*, 3*C*) suggests that the -3p strand could have a more prominent role in regulating neuronal function compared with its -5p counterpart, making the guide and passenger designation somewhat arbitrary in neuronal physiology. This assertion is supported in a previous report demonstrating that miR-9-3p, but not -5p, was involved in the regulation of hippocampal memory and synaptic plasticity (Sim et al., 2016).

Another novel finding of this study was the identification of distinct protein binding partners for miR-9-5p and miR-9-3p (Fig. 7*C*), and to our knowledge this study is the first to identify proteins associated with these mature miRs and their shorter degradation products. Eukaryotic translation initiator 5 (EIF5) was one identified protein that was associated with the full-length miR-9-5p, but not miR-9-3p, in the vHIPP (Fig. 7*C*). EIF5 has been shown to interact with EIF1A via its C-terminal domain (Luna et al., 2013), and EIF1A is a recently discovered component of the RNA-induced silencing complex (RISC; Yi et al., 2015). These data raise the possibility that an EIF1A:EIF5 complex could be associated directly, or indirectly, with miRs that are loaded onto RISC. Another interesting protein that was identified in the vHIPP was tRNA ligase, as it was shown to only associate with the approximated miR-9-5p degradation product (16 nT; Fig. 7*D*). Recently, tRNA fragments have been shown to be loaded onto AGO and even exert gene regulatory functions (Kuscu et al., 2018), suggesting potential crosstalk between the molecular pathways of tRNA and miR degradation products.

The importance of the proteins that we found associated with the shorter miR fragments (i.e., the approximated degradation products) is unclear. The proteins appeared to be specific to each size product (16 vs 12 nT), and the total number of associated proteins were reduced as the nucleotide sequence was progressively shortened (Fig. 7*C–E*). Direct and specific protein binding would suggest that these miRs could be functional and potentially bind to the same targets as the full-length parent, given that the entire 8-nT seed sequence remained intact. There is no evidence that these short fragments can bind to AGO proteins making it unlikely that any functional effect would be similar to the full-length miR; however, these highly stable short fragments could block the ability of a full-length miR to bind to its mRNA target through competition at the seed sequence. The shorter degradation products were much more stable than the full-length miRs, and one possibility is that the associated proteins are important for maintaining the stable expression of these fragments. However, it is important to consider that due to the crosslinking reaction required for immunoprecipitation, the identified proteins represent only those that were in close proximity with the biotinylated miR of interest and do not necessarily reflect direct binding.

The myriad of molecular factors contributing to the rapid degradation of miRs in neurons have still not been fully elucidated. One possibility is that neuronal miRs are partially regulated by the process of TDMD (Park et al., 2017; Bitetti et al., 2018; Ghini et al., 2018; Kato, 2018; Wightman et al., 2018). Indeed, it has been reported that TDMD occurs to a higher degree in primary neurons compared with fibroblasts (de la Mata et al., 2015), but it remains to be seen if RNA-mediated degradation of miRs can fully explain rapid turnover kinetics. Our data showed that elimination of the cellular proteins, but not RNAs, through pre-digestion with proteinase K was sufficient to stabilize the normally rapidly degraded miRs for up to 4 h (Fig. 4*A*,*B*), raising the possibility that the primary contribution of other cellular RNAs is to recruit the proteins required for miR degradation.

Our data using brain tissue lysate would suggest that miR-9-5p and miR-9-3p are degraded on a seconds-to-minutes time scale, respectively. However, Winter and Diederichs (2011) showed that the expression of AGO proteins was critical in determining global miR stability, and we estimated that <10% of our input miR were efficiently loaded onto AGO2 (Extended Data Fig. 1-1). Importantly, the loading efficiency was similar for both miR-9-5p and miR-9-3p, suggesting that the relative stability of miR-9-3p was the result of differential degradation. However, it is difficult to estimate how much endogenously expressed miR-9 is loaded onto AGO2 at any given time, and these numbers have not been reported to our knowledge. Nevertheless, we expect that the degradation kinetics reported in this study are likely reflective of the mature miR after it has unloaded from the RISC, thus leaving the single stranded transcript exposed to the cytoplasmic milieu. Notably, Baccarini et al. (2011), reported that a single miR can be recycled to have multiple mRNA targets, leaving the intriguing possibility that mature miRs can be reloaded onto AGO proteins after the completion of their initial RNAi function. Nevertheless, our data provide valuable information about the *cis* features of miR-9-5p and miR-9-3p that contribute to differential stability. This modified assay (Chatterjee and Grosshans, 2009) used physiologically relevant levels (10 fmol) of miRs, in the context of cellular factors that are endogenously present in neurons, thereby allowing us to assess inherent sequence and/or structural features of each miR that contributes to stability. Our data clearly demonstrate that in brain tissue, each miR exhibited different rates of degradation and resulted in different cleavage products, which were also brain-region specific, despite being subjected to identical experimental conditions. While it is still unclear which enzyme was responsible for the degradation that we observed, it is evident that miR-9-5p degradation was directly correlated with total protein concentration in the lysate based on our dilution experiments and digestion of proteins with proteinase K (Fig. 4*A*,*B*). Furthermore, the rapid degradation that was observed using tissue lysate suggests that a unique protein is present in brain tissue lysate compared with homogenous neuronal cell lines, as protein concentration was held constant for both experiments. It is also possible that extracellular RNases could have contributed to the acceleration of the decay kinetics. However, our experiments showing that there were brain-region-specific differences suggest that the majority of degradation was most likely due to region-specific expression of intracellular proteins that were critical for differential stabilization and not due to extracellular RNases. In addition, miR-9-5p was relatively stable in primary astrocytes (Fig. 5*B*,*C*), suggesting that the enzyme responsible for rapid degradation might be unique to neurons or have relatively lower expression in astrocytes.

Previous studies have used transcriptional inhibition, such as with actinomycin D, followed by qPCR to profile degradation kinetics of mature miRs (Sethi and Lukiw, 2009). Our own results from transcriptional inhibition studies indicate that miR-9-3p was again more stable than miR-9-5p under those conditions; in fact, miR-9-3p levels remained stable throughout all the time points that were measured (Fig. 2). While these studies are beneficial in that turnover kinetics can be measured in intact cells, transcriptional inhibition has been repeatedly shown to negatively affect cellular growth and survival. Therefore, it remains unclear how the turnover kinetics in these altered physiological contexts reflect those of native cells. Recent approaches using metabolic labeling can circumvent many of the obstacles posed by transcriptional inhibition (Marzi et al., 2016). In those experiments, 4-thiouridine, a nucleoside analog, is added to the cellular growth medium and incorporated by nascent RNAs that are actively transcribed. This analog is often modified by biotinylation so that it can be efficiently isolated from the endogenous pool of RNA. The purified transcripts are then quantified by qPCR or other sequencing methods to determine the steady state of the specific RNA of interest. However, due to the intrinsic disparity in the uridine content of miR-9-5p and miR-9-3p, ∼48% and ∼18%, respectively, the purification efficiency of biotinylated 4-thiouridine in each of these miR transcripts is also likely to be different, making it difficult to compare their rate of degradation. In fact, Rädle et al. (2013) concluded that short RNA species with low uridine content are likely to escape biotinylation-mediated precipitation even at high 4SU starting concentrations.

Although metabolic labeling might best approximate the half-lives of miRs under normal cellular conditions, it does not easily allow for biochemical manipulations that are necessary to parse out both *cis* and *trans* factors of a specific RNA that could be important for determining its degradation kinetics. For instance, our data indicated that simply altering three nucleotides at the 3′ end was sufficient to alter the degradation kinetics of both miR-9-5p and -3p to an intermediary degradation rate (Fig. 6*C*), suggesting that the 3′ end of mature miR-9 represents a critical motif for protein interaction. Indeed, multiple reports from the literature suggest that the 3′ end of miRs are an essential element. For example, the 3′ terminal seven nucleotides of miR-382 were observed to be necessary for its rapid decay (Bail et al., 2010). Furthermore, the terminal 3′ nucleotide has been shown to be monoadenylated by GLD2 causing a stabilizing phenotype in a subset of miRs expressed in human fibroblasts (D'Ambrogio et al., 2012); however, GLD2-catalyzed monoadenylation of miRs had no stabilizing effect in the hippocampus, providing further evidence of tissue-specific regulatory pathways (Mansur et al., 2016). In *Arabidopsis thaliana*, a 3′ to 5′ exoribonuclease (Atrimmer) has been shown to be critical for miR turnover (Wang et al., 2018), whereas the 3′ to 5′ exoribonuclease, DIS3L2, was observed to degrade miRs in mammalian cell lines (Haas et al., 2016), further supporting the notion that the 3′ terminus can serve as a critical recognition element in miR processing and degradation. Interestingly, our data showed that the 5′ uracil of miR-9-5p was also important for conferring stability, as removal of this nucleotide, representing an endogenous miR-9-5p isomiR, resulted in enhanced stability compared with the canonical sequence (Fig. 6*D*). This finding is interesting in the context of recent reports demonstrating that this miR-9-5p isomiR can be generated by alternative Drosha cleavage of primary miR-9 (Bofill-De Ros et al., 2019). This isomiR shifts the seed sequence of the miR, thereby expanding the scope of its respective mRNA targets. Functionally, it was identified that prevalence of this miR-9-5p isoform correlated with tumor progression in low-grade glioma (Bofill-De Ros et al., 2019), suggesting that its inherent stability could contribute to unhinged, and even detrimental, downstream consequences in neural pathophysiology. Our degradation assays showed that little, if any, degradation of miR-9-5p and -3p occurred at the 5′ end, based on total densitometry of all bands, including the smaller degradation products, between the initial to the final time point. However, Meziane et al. (2013, 2015) showed that the decapping enzyme DcpS was critical in determining the degradation kinetics of miRs by recruiting the 5′ to 3′ exoribonuclease XRN2 to the processing site in both *Caenorhabditis elegans* and in human model systems, allowing for the possibility of XRN2 is a contributor of 5′-initiated miR-9-5p and -3p degradation. These *cis* contributions of even single nucleotides in altering degradation kinetics propose the exciting possibility that other neuronal miRs, which vary in their nucleotide compositions, may all exhibit unique degradation profiles in the CNS.

Overall, we have shown that miR-9-5p degradation kinetics are likely very rapid in the intact rat brain, consistent with previous studies. However, miR-9-3p (passenger) degradation had not been previously characterized, and our data indicate that the passenger strand is more stable than the guide in neuronal cells, suggesting that it could have a more prominent role in the regulation of neuronal physiology. Furthermore, we provide evidence that the differential degradation kinetics of miR-9-5p and miR-9-3p can be explained, in part, by both *cis* and *trans* elements, underscoring the complexity of miR degradation in the CNS.

## Synthesis

Reviewing Editor: Margaret McCarthy, Univ of Maryland School of Medicine Decisions are customarily a result of the Reviewing Editor and the peer reviewers coming together and discussing their recommendations until a consensus is reached. When revisions are invited, a fact-based synthesis statement explaining their decision and outlining what is needed to prepare a revision will be listed below. The following reviewer(s) agreed to reveal their identity: Christopher Morgan.

In this ms, the authors showed that the differential degradation kinetics of the neuronally enriched miR-9-5p (guide) and miR-9-3p (passenger) that are derived from the same RNA duplex. miR-9-5p degradation kinetics are quite rapid in the brain, whereas miR-9-3p is more stable than miR-9-5p in neuronal cells, which had not been previously characterized. Furthermore, they identified, by sequence swapping and mass spectrometry, unique regulatory cis elements positioned at the 3’ nucleotides and trans elements that could contribute to the distinct miR-9-5p and miR-9-3p stability in neurons. The manuscript is well written and the data interpretation is sound.

miRNA are fundamental actors in the regulation of gene expression, providing a layer of post-transcriptional control that complements more traditional transcriptional regulatory mechanisms. Though much attention has been paid to miRNA transcription, processing, and their functional consequences on target mRNA, less is known about how they are cleared from the cell. As these processes could serve as novel mechanisms to regulate gene expression, fulfilling a similar role for miRNA as miRNA do for mRNA targets, the current manuscript should be an important addition to field.

The investigators are assessing the relative degradation kinetics of miR-9-5p and miR-9-3p, and the underlying factors/mechanisms responsible for any strand-specific differences. Most of their experiments involve tracking the levels of exogenous labeled miR-9-5p or miR-9-3p strands and degradation products in neuronal lysates. These lysates were prepared from either relatively homogeneous cultured cells or heterogeneous tissue punches from specific brain regions.

The principal findings are: 1) miR-9-3p is more stable than miR-9-5p in both their cell/tissue lysates (exogenous spike-in miR-9) and in IVB cells (hypothalamus-derived) treated with a transcriptional inhibitor (endogenously expressed miR-9). 2) The stability of both miR-9-5p and miR-9-3p may be dependent on brain region (source of lysate for the in vitro assay). 3) One of the factors driving miR-9-5p degradation is likely a protein (dependent on protein concentration and inhibited by proteinase K pretreatment of lysates). 4) The relative stability of the two miR-9 strands is partially dependent on their 3’ terminal sequence (3 terminal nucleotides).

The authors used an elegant approach to investigate an understudied, but likely important process

1) Statistical analyses appears to be sound. However, why are some reactions displayed as relative densitometry and other times as half-life?

2) Is the degradation curve fit to miR-9-3p in the vHIPP correct (Fig 3b)? It seems a little off given the 15 min time point.

3) It would be helpful to provide more information about the significance of miR-9? Why did you pick this miRNA? How relevant are these data to the regulation of other miRNA

4) It would be useful to address why aged rats were used (not just in the methods).

5) Have the authors ever observed elongated species of miR-9 strands in your degradation assays? Would these be expected given the tailing/trimming phenomenon described as part of the emerging TDMD mechanisms?

6) Although functional miRNA loaded in Ago2 can be found extracellularly, most miRNA action occurs intracellularly (presumably). Given the rapid degradation seen in your tissue lysates (relative to cultured cell lysates), are you concerned that this is driven by extracellular RNases, which might not be physiologically relevant?

7) As the authors state, only a fraction of the labeled miRNAs in your degradation assays was associated with Ago2; in other words, these miRNA are seem to be in excess. Could it be the observed rapid degradation is driven by the “unbound” fraction? Have you tried lower concentration of labeled miRNA substrate?

8) Given you are using oligos/mature miRNA, it is important to address in more detail whether the observed strand-specific stability is due to differences in loading efficiency (in RISC complex) vs. differences in active degradation

9) The authors should check the organization/completeness of their bibliography. On page 3 they cite “Sim et al., 2016” and “Richner et al., 2015”. The first is not listed alphabetically and the second reference is not in the bibliography.

10) A minor concern to improve the manuscript is that the authors may need more introduction about neuronal activity-dependent regulation of microRNAs in the nervous system. For example, see Sim SE, et al. Mol Cells. 2014. 37(7):511-517.

## Author Response

We thank the reviewers for their time and thoughtful suggestions to improve this manuscript. The manuscript has been revised (see manuscript with tracked changes), and the specific response to each of the comments is noted below.

1) Reviewer Comment: Statistical analyses appears to be sound. However, why are some reactions displayed as relative densitometry and other times as half-life?

Author Response: Relative densitometry was analyzed for all radioactive signals between samples at each time point. The Origins Software program was also used to calculate half-lives and exponential decay functions based on the collective 5 time points. There were some instances where the degradation kinetics were so rapid that it was impossible to accurately calculate the half-life; therefore, we could not discriminate between constructs. In such instances, it was more appropriate to analyze the differences between constructs based on their relative densitometry at each time point. This allowed us to differentiate between degradation kinetics (i.e. slope over time) and half-life.

2) Reviewer Comment: Is the degradation curve fit to miR-9-3p in the vHIPP correct (Fig 3b)? It seems a little off given the 15 min time point.

Author Response: The exponential decay function for miR-9-3p in the vHIPP (Fig. 3B) is correct. The curve represents a “best fit” function that takes in to consideration all 5 time points, as mentioned in comment #1. Importantly, the same fitting parameters were used across all scatterplots.

3) Reviewer Comment: It would be helpful to provide more information about the significance of miR-9? Why did you pick this miRNA? How relevant are these data to the regulation of other miRNA.

Author Response: We have included more information concerning the functional significance of miR-9 in the Introduction. To our knowledge, these studies are the first in its kind to examine the degradation kinetics of miRs using brain tissue lysate; therefore, it is difficult to extrapolate the relevance of these data to the degradation of other neuronal miRs.

However, in support of the finding that cis elements are crucial for determining the degradation kinetics of neuronal miRs, we have unpublished results illustrating that different miRs exhibit unique half-lives in cell lines (IVB).

4) Reviewer Comment: It would be useful to address why aged rats were used (not just in the methods).

Author Response: Aged rats were used for these studies because previous reports observed increased age-related expression of miR-9 in adult rats. Therefore, we wanted to investigate the kinetics of miR-9 turnover at an age where it was likely to be functionally relevant. This rationale has now been added to the Introduction.

5) Reviewer Comment: Have the authors ever observed elongated species of miR-9 strands in your degradation assays? Would these be expected given the tailing/trimming phenomenon described as part of the emerging TDMD mechanisms?

Author Response: This is a very interesting idea and might have been anticipated. However, we did not observe any elongated species in our system, perhaps because tailing mechanisms are initialized upon hybridization with an mRNA target. It is possible that the neuronal miRNAs in our system either degraded before they could hybridize with their target mRNA or the trimming/tailing mechanisms require an intact cellular environment.

6) Reviewer Comment: Although functional miRNA loaded in Ago2 can be found extracellularly, most miRNA action occurs intracellularly (presumably). Given the rapid degradation seen in your tissue lysates (relative to cultured cell lysates), are you concerned that this is driven by extracellular RNases, which might not be physiologically relevant?

Author Response: The observation that there were brain-region specific differences suggest that it is unlikely the rapid degradation was primarily driven by extracellular RNases, because we would expect similar levels of extracellular RNases in all of these samples (Fig. 3C). Therefore, it is reasonable to conclude that the intracellular components of specific brain regions (vHIPP vs. SON) were responsible for determining the differential degradation kinetics of miR-9. Additionally, the relative stability of miR-9-3p compared to miR-9-5p was observed in both tissue and cell culture lysates. If rapid degradation was primarily driven by extracellular RNases, we would expect that miR-9-3p might also be rapidly degraded with no distinction to miR-9-5p. However, we cannot preclude the contribution of extracellular RNases and have included this possibility as a limitation of our experiment in the Discussion.

7) Reviewer Comment: As the authors state, only a fraction of the labeled miRNAs in your degradation assays was associated with Ago2; in other words, these miRNA are seem to be in excess. Could it be the observed rapid degradation is driven by the “unbound” fraction? Have you tried lower concentration of labeled miRNA substrate?

Author Response: As we mention in the Discussion, we expect that the degradation kinetics reported in this study are likely reflective of the mature miR after it has unloaded from the RNA-induced Silencing Complex (RISC), thus leaving the single stranded transcript exposed to the cytoplasmic milieu. Notably, Baccarini et al. reported that a single miR can be recycled to have multiple mRNA targets, leaving the intriguing possibility that mature miRs can be reloaded onto AGO proteins after the completion of their initial RNAi function (2011). However, we do not think the rapid degradation was because the miRs were in excess. Rather, single stranded oligonucleotides (such as the constructs used for these experiments) have a lower efficiency of loading to Ago2 compared to the double stranded miRNA precursor. Similarly, the stoichiometry of endogenous miRNA abundance exceeds the cellular levels of Ago2. The miR:protein ratios used for our experiments were adapted from a previous publication (Chatterjee & Helge Großhans, 2009), which used these same ratios based on endogenous miR levels in the femtomolar range. We tested ratios within the range indicated in the previous publication and used the one that provided the best signal above the limit of detection.

8) Reviewer Comment: Given you are using oligos/mature miRNA, it is important to address in more detail whether the observed strand-specific stability is due to differences in loading efficiency (in RISC complex) vs. differences in active degradation

Author Response: This is a very important distinction and we have added that point to the Discussion. We have shown that the loading efficiency is similar for both miR-9-5p and miR-9-3p; therefore, we think that the observed strand-specific stability is the result of active degradation.

9) Reviewer Comment: The authors should check the organization/completeness of their bibliography. On page 3 they cite “Sim et al., 2016” and “Richner et al., 2015”. The first is not listed alphabetically and the second reference is not in the bibliography.

Author Response: This has been corrected.

10) Reviewer Comment: A minor concern to improve the manuscript is that the authors may need more introduction about neuronal activity-dependent regulation of microRNAs in the nervous system. For example, see Sim SE, et al. Mol Cells. 2014. 37(7):511-517.

Author Response: We agree this is an important aspect to consider when investigating neuronal miRs and have added more in the introduction using the citation, as suggested. However, we did not elaborate extensively because of the word limitations for the Introduction and our inability to experimentally address this unique aspect in our system.
